# Collaborating constructively for sustainable biotechnology

**DOI:** 10.1038/s41598-019-54331-7

**Published:** 2019-12-13

**Authors:** Nicholas E. Matthews, Carrie A. Cizauskas, Donovan S. Layton, Laurence Stamford, Philip Shapira

**Affiliations:** 1 Manchester Institute of Innovation Research, Alliance Manchester Business School, The University of Manchester, Booth Street West, Manchester, M15 6PB UK; 2 School of Chemical Engineering and Analytical Science, The University of Manchester, The Mill, Sackville Street, Manchester, M1 3AL UK; 3 Manchester Synthetic Biology Research Centre for Fine and Speciality Chemicals, Manchester Institute of Biotechnology, The University of Manchester, 131 Princess Street, Manchester, M1 7DN UK; 4 Zymergen Inc., 5980 Horton St #105, Emeryville, CA 94608 USA; 5 School of Public Policy, Georgia Institute of Technology, Atlanta, GA 30332-0345 USA

**Keywords:** Biopolymers, Environmental impact, Environmental social sciences, Sustainability

## Abstract

Tackling the pressing sustainability needs of society will require the development and application of new technologies. Biotechnology, emboldened by recent advances in synthetic biology, offers to generate sustainable biologically-based routes to chemicals and materials as alternatives to fossil-derived incumbents. Yet, the sustainability potential of biotechnology is not without trade-offs. Here, we probe this capacity for sustainability for the case of bio-based nylon using both deliberative and analytical approaches within a framework of *Constructive Sustainability Assessment*. We highlight the potential for life cycle CO_2_ and N_2_O savings with bio-based processes, but report mixed results in other environmental and social impact categories. Importantly, we demonstrate how this knowledge can be generated collaboratively and constructively within companies at an early stage to anticipate consequences and to inform the modification of designs and applications. Application of the approach demonstrated here provides an avenue for technological actors to better understand and become responsive to the sustainability implications of their products, systems and actions.

## Introduction

Recognising the growing call for more environmentally, economically, and socially responsible societies, emerging technologies are increasingly promoted on the promise of sustainability benefits. Synthetic biology, a sector that integrates engineering principles and computational approaches with advances in biological techniques, is often advocated as an example of a field that is broadly developing more sustainable solutions^1^. By enabling biological routes for the production of a wide range of fuels, chemicals, and materials from biomass, synthetic biology could displace existing fossil-based production routes with renewable alternatives^2,3^. Given their potential, it would seem appropriate to harness such technologies to help deliver greater sustainabilty^4^.

However, sustainable development is highly complex, presenting issues that span both social and natural domains and which have characteristics of interrelatedness, uncertainty, and incommensurability^5^. The UN’s sustainable development goals (SDG) articulate but hardly simplify this complexity, outlining seventeen broad and interrelated goals^6^. Concepts and practices of sustainability remain subject to diverse interpretations. As a result, while there is increasing recognition of the urgent need for wide ranging sustainability transitions, there remains limited agreement on how this should be undertaken and what this should achieve.

How can we navigate through this complexity and promote the sustainable development of emerging technologies? A growing sustainability literature emphasises the need for an open-ended approach characterised by experimentation and learning; this body of literature also recognizes that traditional, top-down “command and control” management and policy approaches to solving such problems are insufficient for robust decision making under conditions of uncertainty^5,7–9^.

Yet, experimentation with sustainable technologies is not simply an exercise in the random sampling of solutions - it must be informed by evidence and supported by continuous, iterative cycles of evaluation and learning^5^. This necessitates the acquisition of knowledge on the sustainability performance and implications of emerging technologies, as well as on the criteria against which they should be judged. Such a process involves evidence gathering from multiple domains and transdisciplinary knowledge generation^10^. To be salient, such evidence must be acquired and integrated into technological design at the early stages of technological development to inform key design decisions before lock-in is established and before further downstream development when change is difficult or costly^11^. This requires the gathering of evidence when limited data is available.

Evidence gathering and experimentation is further complicated by the fact that emerging technologies like synthetic biology are developed and applied largely by and within companies. Traditionally, the role of a company is to maximise financial return while complying with its legal and contractual responsibilities. Companies are also constrained to working within existing systemic frameworks, such as the agricultural sector that provides fermentation feedstocks. Concepts such as the triple-bottom line (TBL) expand this view, and a growing literature explores how companies can simultaneously achieve benefits for people, planet, and profit^12–14^. However, this outlook potentially restricts experimentation with sustainability-orientated innovations to those that are compatible with (short-term) profit^15^. A possible solution is found through promoting responsible research and innovation (RRI)^16^. RRI provides a framework through which companies might assume greater responsibility for the impacts of the innovations they generate, both positive and negative^16^. However, in addition to exploring a (re)distribution of responsibilities for innovation amongst companies and other technological actors (such as governments, regulators and civil-society organisations)^17^, research is needed to strengthen the capacities of companies to engage with the complex socio-technical systems within which they operate^18^.

Clearly, governing and promoting emerging technologies in such a way that they can contribute to sustainable development is no simple endeavour. In this article, we demonstrate how a constructive approach to assessing sustainability can productively grapple with these challenges through a) close collaboration between interdisciplinary researchers and technology actors (in this case, a biotechnology company); b) the application of life-cycle assessment methodologies at the conceptual design stage under high uncertainty; and c) the use of deliberative workshop formats to consider sustainability concepts and implications and explore options.

## The Case for Constructive Sustainability Assessment

Members of our group have previously outlined a Constructive Sustainability Assessment (CSA) approach to navigating through the complexity of assessment and governance of emerging technologies towards sustainability^19^. Conceptually, we draw on frameworks for deliberative and constructive technology assessment and governance to articulate four key design principles for constructive sustainability assessment:

- Design principle 1: Mobilise transdisciplinarity to allow knowledge generation across multiple domains and integration of findings into decision-making.
- Design principle 2: Implement tentative and incremental governance in order to keep technological options open^20,21^.
- Design principle 3: Propagate and explore uncertainty as a core feature of the assessment exercise.
- Design principle 4: Anticipate potential future impacts of emerging technologies in terms of sustainability.

**Figure 1:**
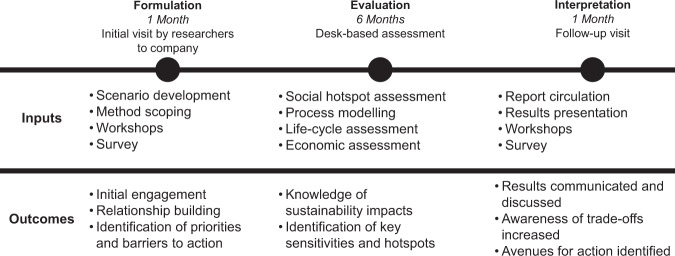
Overview of the CSA process undertaken for this study.

A methodological framework for operationalising these design principles follows a three-step approach (Fig. 1). The *formulation* stage involves deliberative workshops and evidence gathering involving stakeholders. The results of these activities inform the sustainability assessments subsequently undertaken during the *evaluation* process utilising established methods such as life-cycle assessment (LCA). In a subsequent *interpretation* stage, the results of the process are then discussed and elucidated during further workshops to deliberatively explore the implications of the results.

CSA is designed to be broadly applicable to emerging technologies and a range of production systems wherein differing analytical approaches may be utilised during the *evaluation* stage within a consistent methodological framework. The approach is also designed to be flexible and scalable according to the time and resources available, such that it could be applied by various technological actors and organisations of differing sizes from start-up to multinational. Here, we demonstrate the operationalisation of CSA in the context of a relatively young (~6 years old) biotechnology company still developing internal practices and processes as well as exploring new markets. Our study’s test company is also involved in developing diverse and interdisciplinary projects and products across multiple scales, presenting an appropriate laboratory for developing and testing CSA methods. Many of the company’s employees have been involved in several different projects at different times during company growth, at different points of scale-up, and across different product types, giving the study a diverse cross-section of industry experiences.

We recognise that techno-economic analyses (TEAs) are already employed by companies to evaluate the economic feasibility of processes. In biotechnology, early application of TEAs is increasingly recognised as important to integrate downstream industrial-scale considerations into the design phase, thereby facilitating smoother scale-up^22^. TEA and CSA both require prospective analysis of anticipated applications, grappling with uncertainty issues to ensure timely acquisition of knowledge. TEA provides a framework for carrying out prospective modelling. CSA expands the scope to consider additional environmental and social parameters while, through its constructive approach, embedding the practice within the management and social structures of the company.

## Results

### Formulating the assessment

To operationalise and illustrate our CSA approach, we established a transatlantic collaboration consisting of a biotechnology company developing fermentation products across multiple scales and uses, and a team of university researchers. The company wanted to better understand the sustainability implications of the bio-based products they develop in engineered microbes. The university researchers were interested in developing new approaches to assess sustainability that could grapple with its subjective nature and generate findings which could be responsibly integrated into decision-making. Underpinning this team was its transdisciplinary nature (Design principle 1) spanning the social and natural sciences, with skills including molecular biology, business and management, sustainability and environmental science, responsible innovation, and ecology.

**Figure 2:**
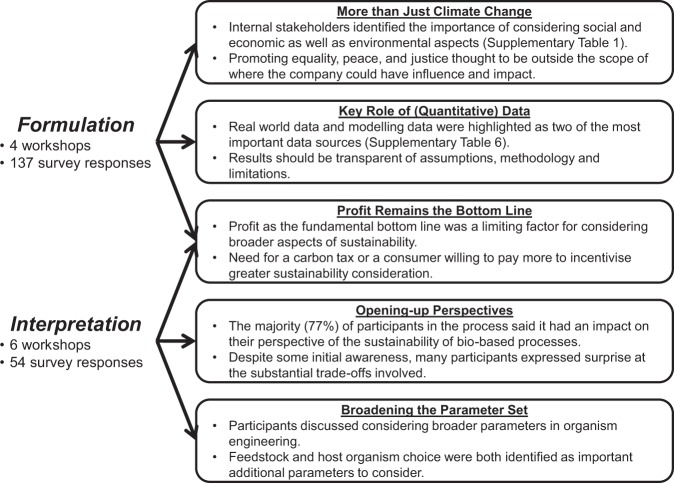
Emergent themes from the *formulation* and *interpretation* stages of the study.

A key feature of the collaboration was the embedding of academic researchers within the company from where they could understand and engage with industry stakeholders and carry out more situated assessments^23^. This began with the *formulation* stage of the assessment, in which internal stakeholders (company employees) were engaged through a survey and workshops (see methods) to discuss questions of what sustainability in biotechnology meant to them and what data formats were useful and informative (See Fig. 2 for emergent themes).

**Figure 3:**
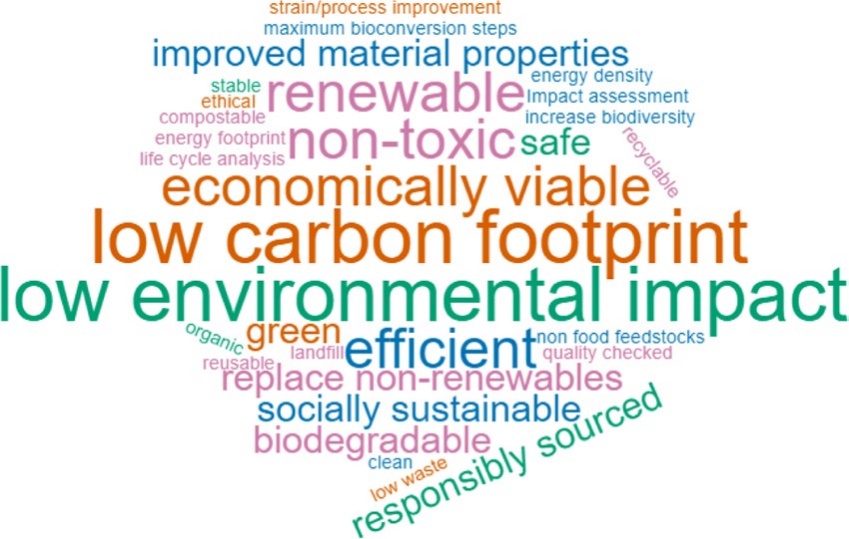
Wordcloud generated from responses in the initial *formulation* workshops to the question: “What characteristics would a sustainable biotechnology product have?”.

We first sought to clarify the sustainability concept employed by the internal stakeholders, exploring the characteristics they felt that a sustainable biotechnology product should have (Fig. 3). Discussions and responses on this topic initially focussed on environmental aspects, particularly on the potential of biotechnology applications to reduce CO_2_ emissions and combat climate change. Being “renewable”, “non-toxic”, and generally “low environmental impact” were also characteristics frequently highlighted as being considered sustainable.

While the initial focus was clearly on environmental impacts, broader notions of sustainability were widely discussed. Beyond tackling climate change (SDG 15) and improving the health of global ecosystems (SDGs 13 & 14), eliminating poverty, hunger, and poor-health (SDGs 1, 2, 3, 6, 7), and sustaining employment and economic growth (SDGs 8, 9, 11) were also seen as areas where biotechnology applications could make positive contributions (Supplementary Table 1). However, the results also highlighted consensus among internal stakeholders that promoting equality, peace, and justice (SDGs 4, 5, 10, 16) was likely to be an effort outside of the influence and capabilities of an individual company. These initial results informed assessments in the subsequent *evaluation* stage, and demonstrated the utility of internal stakeholders’ perspectives for broadening focus. As such, they represented an often-untapped method of encouraging a more open and deliberative approach to innovation within companies (Design principle 2).

While internal stakeholders were keen that the products they developed should yield sustainability benefits across a broad range of dimensions, they also highlighted the need for economic viability; financial constraints ultimately frame the extent of integration of broader elements. This discussion highlighted the importance of undertaking analysis of costs alongside environmental and social assessments, and so we added minimum selling price (MSP) to the subsequent *evaluation* phase as a key parameter.

The *formulation* stage included discussion of methodological aspects to ensure the outputs of the *evaluation* stage were salient for stakeholders. This also allowed the researchers to understand backgrounds and expertise of the various internal stakeholders. For example, there were markedly differing levels of exposure to quantitative methods across employee departments, emphasising that the results of the *evaluation* must be presented in a manner that all can understand. Stakeholders also highlighted the importance of sensitivity testing, use of “real-world” data where possible, and clear articulation of all assumptions. Overall, the *formulation* stage of the CSA approach demonstrated a number of important outcomes:

- Engaging those who might act upon results in the assessment process early-on, achieving trust in and commitment to the process.
- Clarifying the sustainability concept employed by internal stakeholders, providing a normative reference point for subsequent assessments.
- Mobilising the viewpoints of internal stakeholders to expand sustainability perspectives.
- Elaborating, at an early stage, perceived opportunities and barriers to actions which might be taken to promote sustainable biotechnology at an early stage.
- Identifying the relative salience of different methodological tools, data sources, and presentation approaches to diverse audiences.

These activities are essential to ensure the *interpretation* stage of the assessment is relevant to internal stakeholders and actors. The knowledge gained also guides and thus provides legitimacy to the subsequent *evaluation* stage.

### Evaluating bio-based nylon sustainability

Evidence collection at the preliminary stages of technology development is crucial to guide informed experimentation with sustainability-oriented innovations. This section reports on the results of the *evaluation* stage in which the sustainability implications of bio-based nylon were anticipated under high uncertainty (Design principles 3 and 4).

The monomers used in the production of nylons are, at present, derived from fossil fuel-based sources. Production of adipic acid, used in nylon 66, yields large quantities of the potent greenhouse gas N_2_O; one study estimated that adipic acid represents 80% of Chinese industrial N_2_O emissions^24^. Given the potentially significant contribution to climate change of adipic acid production, and the importance of nylon as a polymer in a wide variety of applications, biologically-based monomers for nylon production are an area of interest, but without commercial application as yet.

Cadaverine (1,5-diaminopentane) and putrescine (1,4-diaminobutane) are diamines which can be used to derive bio-based alternatives to nylon through polymerisation with dicarboxylic acids^25^. The biochemical production of both molecules has been demonstrated in *Escherichia coli*^26,27^. Putrescine can be combined with adipic acid to form nylon 46, while polycondensation of putrescine or cadaverine with sebacic acid (from castor beans) yields nylon 410 or nylon 510 respectively^28^. Nylon 510 has been found to have comparable physical properties to the currently predominant nylon 66 and nylon 6^29^.

The collaborative approach described in the previous section allowed the crucial exchange of data and knowledge to enable and guide the assessment of sustainability implications across social, environmental, and economic criteria (see methods). In doing so, we followed an approach similar to anticipatory LCA, whereby uncertainty becomes a fundamental feature of the analysis and is propagated and explored throughout (Design principle 3)^30^. We considered four feedstock scenarios for sugar production, from three geographical locations:

- Glucose and xylose generated from corn stover in the U.S.
- Glucose from corn starch, also in the U.S.
- Sucrose from sugar beets in France.
- Sucrose from sugarcane in Brazil.

Due to a combination of constraints from limited data availability and the nature of the issues at hand, levels of analysis had to be tailored to the sustainability pillar investigated:

- **Social:** Biomass and biorefinery sectors were compared to petrochemicals across the geographical locations considered. This was complemented by a literature review of social issues in the biomass sector.
- **Economic:** Minimum selling price (MSP) was calculated for individual bio-based monomers (cadaverine and putrescine).
- **Environmental:** Comparisons were made across four types of nylon – bio-based nylon 510, 410 and 46 compared to fossil-based nylon 66.

#### Identifying social risks at an early stage

**Figure 4:**
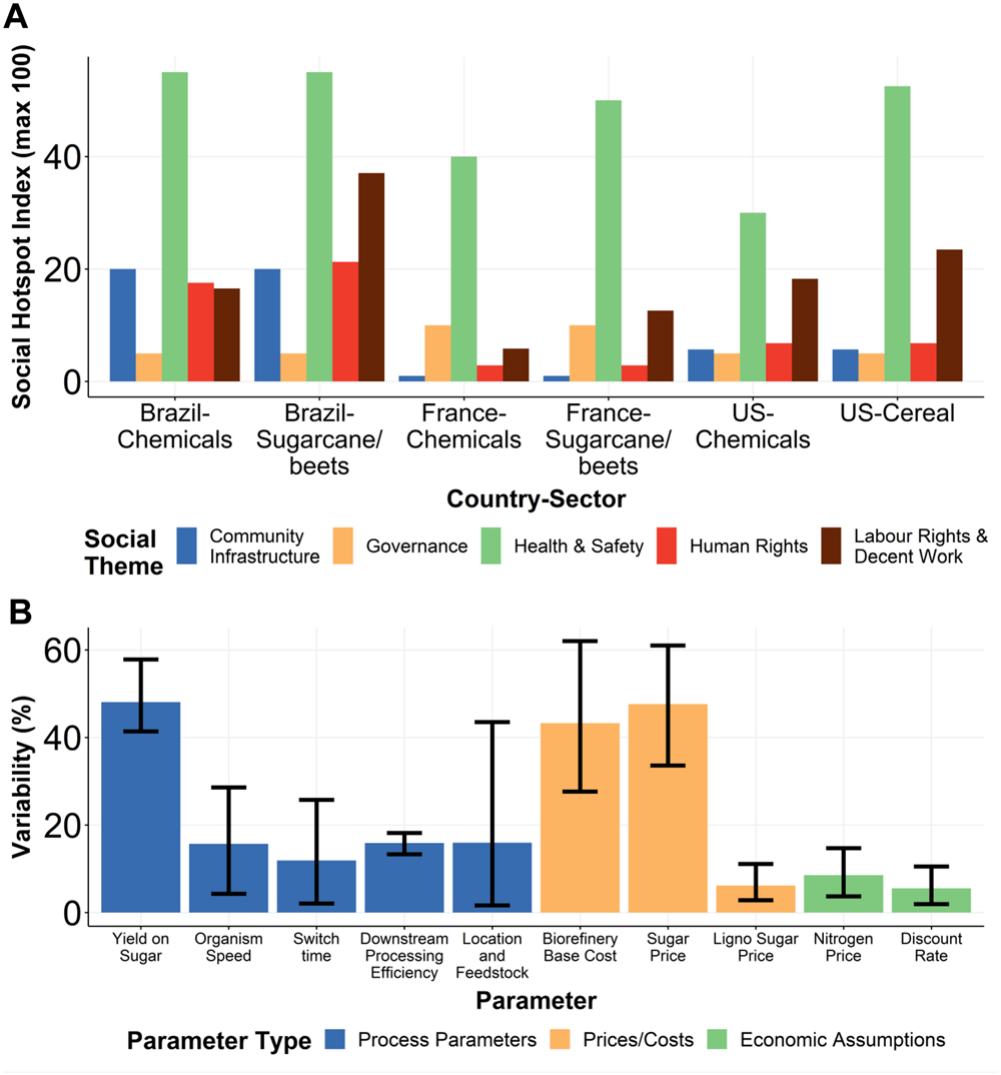
Economic costings and social hotspot results. (**A**) Social hotspots index results for the three geographical scenarios. (**B**) Key parameters affecting minimum selling price (results for putrescine). Only parameters with average sensitivity of greater than 5% are shown. Error bars show 95% confidence intervals from multi-start sensitivity analysis.

We used the social hotspot index (SHI) approach with the social hotspot database (SHDB) to measure potential social risks of bio-based nylon production (see methods)^31^. We calculated the SHI for the country-specific sector (CSS) corresponding to the relevant agricultural sector for each geographical feedstock scenario (Supplementary Table 2). We used the “Chemical, rubber and plastic products” sector as a proxy for biorefineries in the absence of a specific CSS. For all CSS considered, risks to human health and safety were the most significant risks associated with bio-based production, while labour rights and work conditions also represented frequently occurring hotspots (Fig. 4A, Supplementary Tables 4 and 5). Concerns have previously been raised about poor working conditions in biomass production, such as health issues due to the practice of burning sugarcane tops^32^.

The shift towards synthetic biology-enabled bio-based production methods also introduces specific considerations not yet captured in the SHDB. On the positive side, biomass production can lead to investment in local economies and generate local employment. In Brazil, one million people are employed in the sugarcane industry with related improvements in job formality, benefits, and salary^33^. However, feedstock production for biotechnology can also result in consolidation of small-holdings and lead to greater mechanisation, disrupting existing land-ownership, land-use rights, and employment patterns^33^.

The production of biomass for biotechnology can also compete with land for food, driving up global food prices and adversely impacting the world’s economically poorest citizens^34^. More recently, there has been increasing focus on biorefineries that make use of waste feedstocks (e.g. corn stover or wheat straw) or lignocellulosic sugars grown on marginal lands (e.g. *Miscanthus*)^35^. However, removing these resources can decrease soil-carbon stores by removing straw that would otherwise be recycled, and may adversely affect the economics and culture of vulnerable rural communities^33,36,37^.

While it is informative to highlight and understand the potential social hotspots of future bio-based products, it is difficult to fully assess cost-benefit trade-offs involved in these disruptive innovations until these technologies achieve widespread adoption, particularly since many of their impacts are likely to be indirect. However, highlighting these issues at the early stage of biotechnological innovation can guide further analyses and data gathering as commercialisation progresses, such as through social auditing of suppliers and commercial partners. These early and ongoing assessments are critical for allowing incremental consideration of social impacts (both positive and negative) during, rather than after, implementation (Design principle 2).

#### Estimating the minimum selling price

MSP was estimated based on collaborative process modelling to determine the potential costs of individual monomer production (see methods and Supplementary Table 21 for full results). We estimated an average MSP of $3.66 per kg for putrescine (range $1.55–$8.80) and $3.67 per kg for cadaverine (range $1.50–$9.00). At the lower end of these ranges, which would represent a “best-case” or optimised set of parameters, the MSP is competitive compared with fossil-based feedstocks; for example, a typical adipic acid selling price is 2.09 $/kg^38^. However, it is worth noting the volatility of these markets based on the crude-oil price, a factor that becomes further complicated by the addition of carbon taxes and/or consumer willingness to spend more for sustainable products (the ‘green premium’).

Global sensitivity analysis using a multi-start approach (see methods) highlighted the key parameters influencing the results (Fig. 4B). For putrescine production, the model was most sensitive to the microorganism’s yield on sugar with a sensitivity of 48.08% (range 39.20% to 61.60%); this parameter is determined by the efficacy of the microorganism and, therefore, can be engineered. Significantly, the yield represents a core design parameter for companies developing new microorganisms for bio-based production. Similar levels of sensitivity were seen for the capital cost of the biorefinery, at 43.26% (range 22.89–69.66%), and the sugar price, at 47.61% (range 24.00–70.34%). Sugar price and biorefinery cost are not directly within the control of companies developing the base technology (the microorganism); however, the influence of these parameters highlights the importance of considering both upstream (in terms of feedstock source and type) and downstream processing parameters in sustainability assessments.

#### Highlighting environmental trade-offs

**Figure 5:**
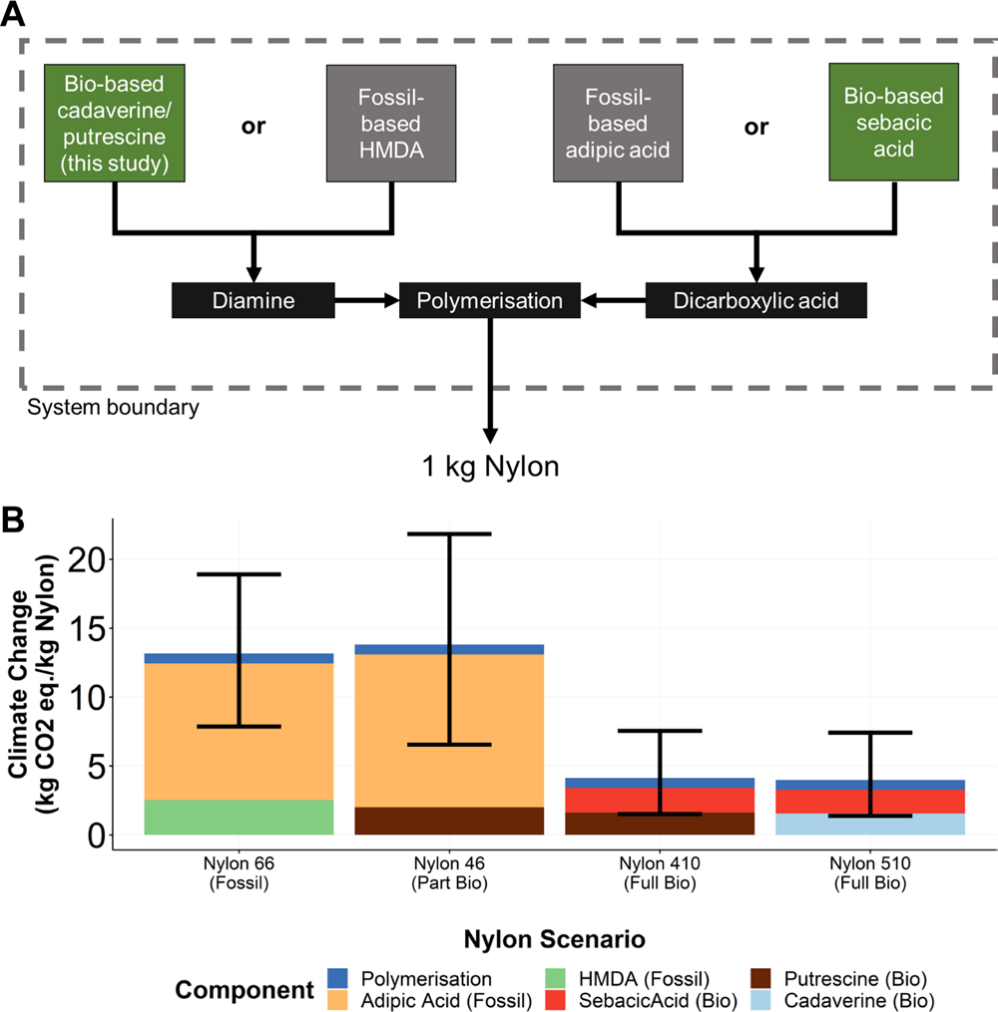
Environmental assessment results (1/2). (**A**) System boundary for nylon comparisons showing how results were combined. (**B**) Climate change impact results coloured by relative contribution of monomers and polymerisation. Error bars show 95% confidence intervals from Monte Carlo simulations.

Using the same process model as for the economic analyses, an LCA was undertaken following the ISO approach (see methods and Supplementary Tables 14–17 for full results)^39,40^. The functional unit for this analysis was 1 kg nylon. Through the combination of published data and the results of analyses carried out in this study (for cadaverine and putrescine), we considered four types of nylon: bio-based nylons 410, 510 and 46 and fossil-based nylon 66 (Fig. 5A, Supplementary Table 12). We found that using bio-based putrescine in the production of nylon 46 resulted in a worse overall climate change impact (more kg CO_2_ eq/kg nylon produced) in 72.45% of simulations compared to producing fossil-based nylon 66 (Fig. 5B): on average the impact of nylon 46 was 3.85% higher than nylon 66 (range of -19.57% to 38.49%). This outcome demonstrates the importance of considering how new bio-based chemicals will integrate into existing supply-chains: 1 kg of nylon 46 requires a greater mass of adipic acid to produce compared to nylon 66, which negates the benefits of replacing HMDA with bio-based putrescine. However, bio-based nylon 410 and nylon 510 both showed superior climate change performance (i.e. lower kg CO_2_ eq/kg nylon produced) in 100% of simulations compared to fossil-based nylon production. We found an average reduction in climate change impact of 64.48% (range of 11.67% to 92.22%) for bio-based nylon 410 and 65.75% (range of 11.75% to 93.05%) for bio-based nylon 510 compared to fossil-based nylon 66.

**Figure 6:**
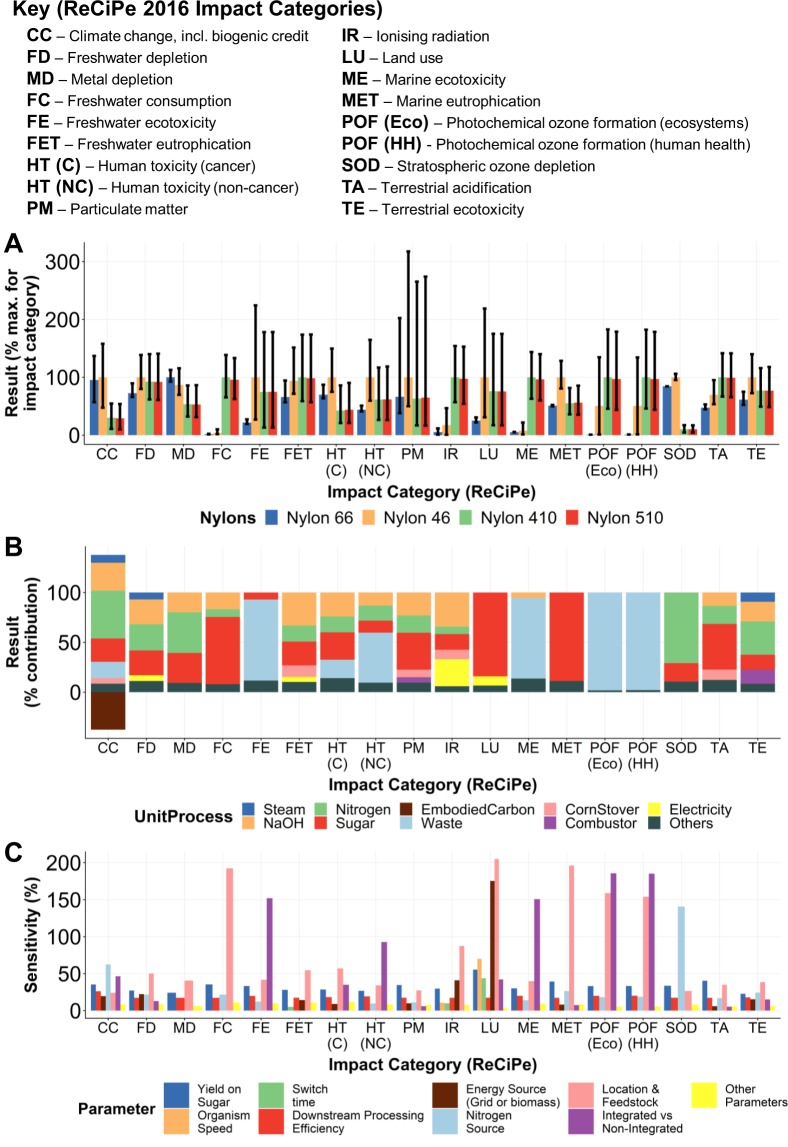
Environmental assessment results (2/2). Error bars show 95% confidence intervals from Monte Carlo simulations. (**A**) Results for all 18 ReCiPe 2016 impact categories across the four nylons considered. Results are normalised by maximum result for each impact category. (**B**) Relative contribution of each background or foreground stage to each impact category result (results for putrescine). Stages contributing less than 5% to each impact category are grouped into the “others” category. (**C**) Influence of parameters on each impact category (results for putrescine). Parameters with an average sensitivity of less than 5% for each impact category are grouped into the “other parameters” category.

Most discussions and assessments surrounding bio-based technologies focus on their ability to reduce net CO_2_ emissions and dependence on fossil fuels^41–43^. However, such a focus risks shifting impacts towards other environmental areas, particularly those involved with land use and agricultural practices required for biomass production for feedstocks. In this analysis, we considered a wider range of environmental impact categories; our results indicated that bio-based nylons generally had worse impacts across a range of impact categories (e.g. freshwater consumption, land-use, freshwater and marine ecotoxicity, terrestrial acidification) compared to traditional fossil-based production (Fig. 6A).

Consistent with design principle 3, we present uncertainties clearly in our results. At this stage of analysis early in the biodesign process, some impact categories contain large uncertainties, leading to somewhat inconclusive results. In other impact categories, we can measure clear differences, demonstrating the utility of carrying out such analyses even at early stages of product development. Furthermore, far from simply representing incomplete knowledge, uncertainties can also highlight areas in which processes can be improved^5^. A combination of hotspot (for influential process stages) and sensitivity analysis (for key parameters) can therefore be highly informative for prioritising efforts to reduce negative environmental and social impacts. In addition, the iterative nature built into the CSA process is designed to both update time-sensitive input data and to reduce algorithmic uncertainties as more data are incorporated into analyses as projects progress.

In our analysis, we identified hotspots in the supply of raw materials such as sugar and nitrogen required by the microorganism, and sodium hydroxide, or other strong alkaline, required for the selected downstream process (DSP) (Fig. 6B). Our multi-start sensitivity analysis indicated that, while microorganism-specific parameters such as yield and productivity influenced the outcomes of all impact categories, parameters relating to feedstocks and DSP were typically even more influential (Fig. 6C). The level of waste handling integration in each process examined was particularly important for determining outcomes of ecotoxicity and photochemical ozone-related impact categories. These results are in contrast to our MSP analysis, in which yield was the most influential parameter. This supports the consideration of parameters beyond yield and productivity when developing sustainable microbe-based biotechnologies.

### Constructive interpretation

Interpreting sustainability assessment results, such as those presented here from the *evaluation* stage, represents a key challenge. In order to support the iterative aspect of CSA and promote the constructive exploration and opening-up of design options by stakeholders (Design principle 2), we pursued a deliberative approach to *interpretation*. We circulated the results of the *evaluation* stage among internal stakeholders through an hour-long company-wide presentation, a summary report, and short (~10 minute) presentations to smaller, departmental workshops in which we then discussed the results. We also distributed a follow-up company-wide survey, similar to our first survey.

Through the deliberative approach we were able to identify what could be responsibly concluded from the results and for which Table 1 provides a summary. The cells of the table encapsulate the results of the analysis described in detail in the previous sections alongside the key uncertainties, sensitivities, outstanding ambiguities, potential routes forward and future actions that were discussed and elaborated during the follow-up workshops. The table demonstrates the kind of rich outputs and findings, spanning a broad range of SDGs, that can be generated through this constructive approach. Ambiguities are inevitable when undertaking analyses at this stage of technological development, but they also suggest areas where further cycles of CSA could clarify or elaborate unknowns.

**Table 1:** Summary of the outcomes of the study as determined through analytical *evaluation* and deliberative *interpretation*.

Sustainability Aspect	Evaluation Results	Hotspots	Key Sensitivities	Ambiguities	Potential Actions
*Tackling Climate Change (SDG 15)*	• Nylon 510/410: Climate change reductions vs nylon 66 • Nylon 46: Climate change increases vs nylon 66	• Biomass (sugar) production • Nitrogen and NaOH • Embodied carbon	• Yield on sugar • Process integration • Nitrogen source	• Future process optimisation • Process parameterisations	• Explore alternative feedstocks • Avoid usage in nylon 46
*Improving the health of global ecosystems (SDG 13*, *14)*	• Increased impact across many impact categories including freshwater and marine ecotoxicity	• Biomass (sugar) production • Nitrogen and NaOH • Waste Handling	• Process integration • Geographical location • Yield on sugar	• Future process optimisation • Process parameterisations	• Explore alternative feedstocks • Greater process integration
*Eliminating poverty, hunger and poor-health (SDG 1*, *2*, *3*, *6*, *7)*	• Growth opportunities for rural areas in global south • Health & safety risks in biomass sector	• Health and safety • Labour rights and decent work	• Geographic location	• Many unknown unknowns • How to measure the fair distribution of costs and benefits	• Engage with value-chain stakeholders
*Sustaining employment and economic growth (SDG 8*, *9*, *11)*	• Potential to displace incumbent fossil-based nylons • Highly optimised scenarios may be able to compete with fossil-based incumbents	• Raw material cost • Base capital cost	• Yield on sugar • Sugar price • Biorefinery base cost	• Cost estimates highly uncertain • Future oil price • Possibility of a green-premium or carbon tax	• Reflective and inclusive dialogues to explore options

Unless otherwise stated, bullet points relate to all bio-based nylon scenarios compared to fossil-based nylon.

For tackling climate change (Table 1, row 1), the use of bio-based putrescene alongside fossil-based adipic acid in nylon 46 should clearly be discouraged. In addressing climate change alongside improving the health of global ecosystems (Table 1, row 2), it was clear from workshop discussions that there would be benefits in considering broader parameters when engineering and optimising organisms. Titre is commonly the key parameter against which new microorganisms and strains are evaluated, but our findings highlighted the importance of also considering yield due to its effect on biomass usage. The significance of biomass production for overall sustainability performance also stimulated discussion regarding the use of alternative feedstock. Flexibility of feedstock remains a key issue when the majority of available sources remain first-generation crops that compete with food production. Use of alternative feedstocks such as waste streams could be achieved through the exploration and engineering of different host organisms and strains which can grow on more sustainable and currently available feedstocks.

Highlighting health and safety risks and potential issues faced by farmers helped us to explore broader social aspects such as poverty, hunger and poor-health (Table 1, row 3). While specific responses to these issues can be more difficult to lock-down given their complex and often macro-level nature, highlighting them at an early stage encourages continued attention through initiatives, such as social auditing of the supply-chain, throughout scale-up and commercialisation. Engagement with potentially affected stakeholders throughout the value-chain from farmers to consumers may help to further explore the complex social implications of a transition to bio-based manufacturing approaches.

Finally, economic ambiguities (Table 1, row 4) are some of the hardest for a company to resolve as they relate to systemic issues that may need to be tackled at a higher level. Deliberation allowed reflection upon how biotechnology companies could tackle these ambiguities given these constraints. The locked-in nature of incumbent fossil-based technologies represents a key barrier to systemic change. In addition to this, the potential benefits of sustainable innovations such as bio-based production relates in a large part to externalities such as carbon dioxide emissions. The internalisation of these externalities, such as through a carbon tax paid by those generating emissions, would generate greater market incentive for sustainable innovation such that the sustainability benefits can become part of a company’s core value-offering. However, issues such as carbon taxes are currently subject to active societal debate. Reflective and inclusive dialogue with governments, clients and competitors, regulators, and wider society could be an appropriate route forward^44,45^.

The intention of a CSA process is to question assumptions, open-up options, and build capacities in anticipation, reflexivity, and responsiveness for the future (Design principles 2 and 4)^44^. In this study, while awareness of sustainability issues surrounding biotechnology applications varied amongst the internal stakeholders, many participants commented on how the data demonstrated the complexity and trade-offs involved, and that “bio isn’t always better”. In a follow-up survey, 77% of respondents stated that the CSA process had at least a small impact on the way they “think about the sustainability of bio-based products” (within which 21% indicated a moderate impact and another 21% indicated a significant impact). The use of anticipatory assessments can therefore be successful in questioning the prior assumptions of stakeholders (Fig. 2) alongside, as discussed above, identifying potential actions and routes forward.

Here, we have carried out a single cycle of CSA focussed on a single application. Wider application and repeated iterations are required to allow further analysis and incremental governance. Internal stakeholders demonstrated appetite for this and emphasised the utility of starting early and “building-up” the process over time. CSA will also need to align to current business practices. Combining the approaches of TEA and CSA may ultimately be the best approach, by simultaneously anticipating sustainability and commercialisation challenges.

## Discussion

In this article, we demonstrate a collaborative and constructive sustainability assessment applied to bio-based nylon production. Empirically, we find that bio-based nylon alternatives have the potential to yield substantial improvements over petroleum-based analogues in terms of climate change, but show equivocal results in several other environmental impact categories, a result that is consistent with those of other published analyses^46,47^. Our results for the current cost of biomaterial production, though uncertain, support the view that while bio-based approaches struggle to compete on a like-for-like basis with established fossil-based incumbent technologies, under optimistic future scenarios favourable economic competitiveness could be seen^48^. Parameters such as feedstock choice, yield-per-organism, and level of process integration are identified as promising areas for improving sustainability performance and highlight the need to consider more than simply yield and productivity to achieve sustainable biotechnology development. Socially, results suggest that particular attention should be given to health and safety risks in biomass production, as well as to potential disruption to local employment and cultural practices when producing feedstocks. This is consistent with findings from Valente *et al*. in a study of the social implications of future biorefineries^49^.

More broadly, our case study demonstrates a promising operationalisation of the CSA approach^19^. Building on the arguments of those who regard internal stakeholders as a potential source of incentives for companies to engage in RRI^50^, we illustrate the utility of mobilising internal stakeholders throughout the process to open up perspectives and embed RRI principles early in the manufacturing process. Application of CSA in an industry context, while bringing its own challenges, is essential to allow these important players to pursue and experiment with sustainable innovation. We add much needed empirical evidence to the growing discussions in the literature regarding how companies can align their practices to sustainability goals and embed responsible innovation^51,52^. The case demonstrated how a relatively new company deployed CSA in an attempt to align practices with sustainability goals, with experiences and insights gained that are applicable to future new product developments. The approach could also be used by more established companies for enhanced alignment of company practices with sustainability.

Crucially, this (re)alignment needs to be part of a two-pronged, multilevel approach^18^. Firstly, companies must consider and manage the impacts of the innovations they create and promote, including both negative and positive effects of products and processes. A CSA approach critically aids companies to understand, anticipate, reflect and act upon these outcomes. Simultaneously, changes are necessary within the broader market environment within which companies operate to favour truly sustainable innovations. While such rearrangements, including honing feedstock production for greater efficiency and suitability to biotechnology needs, have to be stimulated at a higher level, enabling companies to foresee these challenges and to inform their own and others’ actions can contribute positively to a sustainability transition.

Scaled up, the results of multiple instances of CSA analyses across several industrial stakeholders would create a large body of biotechnology sustainability data and experience able to inform public-private partnerships and policymakers in efforts to undertake systemic changes not achievable by individual actors. This could be through greater attention to evidence-based sustainability in research funding, design and evaluation, feedstock development and use, modelling, training, regulatory review, and road mapping.

## Methods

This study followed the methodological approach for Constructive Sustainability Assessment (CSA) outlined in a previous publication^19^. In this case study, we completed one cycle of CSA. This involved three stages: *formulation*, *evaluation*, and *interpretation*. The study received ethical approval from the Alliance Manchester Business School ethical review panel.

### Formulation

During the *formulation* stage, we conducted four, hour-long workshops that engaged a total of twenty company employees from four departments (Supplementary Table 6). N.E.M. facilitated all workshops. The workshops explored the following topics:

- What does it mean for a product to be sustainable? What aspects matter?
- In terms of sustainability, what sources and types of information are useful and influential?
- What kinds of data and presentation formats are preferred?

During the workshops, participants were asked to electronically submit answers to the question: “What characteristics would a sustainable biotechnology product have?” The answers were cleaned to combine similar terms and used to produce the word cloud in Fig. 3.

In addition, we circulated a survey electronically to all company employees, and received 137 full responses. The company had ~500 employees at the time of surveying. The survey covered the following topics:

- The significance of different aspects of sustainability for the biotechnology sector.
- Preferred data sources.
- Personal sustainability motivations.

Text and notes from the survey and workshops were coded and analysed. Based on these outputs as well as discussions within the team, we developed the subsequent *evaluation* stage. This involved primarily the selection of indicators, methods, and scenarios.

### Evaluation

We selected cadaverine and putrescine as bio-based targets of interest as they can be used as precursors to make useful chemicals and materials such as nylons. The goal of the *evaluation* stage was to assess the sustainability implications of using bio-based cadaverine and putrescine for the production of nylon compared to fossil-based alternatives (nylon 66). The scope of the study is articulated individually for each of the assessment stages.

#### Social assessment

This study made use of the social hotspot risk mapping tool, an online interface to the social hotspots database (SHDB) which provides data on social risks to the resolution of country specific sector (CSS)^31^. This database uses more than 50 indicators, both quantitative and qualitative, to characterise 5 social categories. The results for each social category can be aggregated for a CSS using the social hotspot index (SHI)^53^. This involves assigning a risk level based on indicator values following which a weighted sum is calculated which is then normalised against the maximum possible weighted sum for that CSS, with a maximum score of 100. The mathematical formula for this is shown below (source: *new earth b*^31^):

$$SH{I}_{cat}=\,\mathop{\sum }\limits_{T=1}^{n}\,({R}_{avg}\,\times \,{W}_{T})\,/\,\mathop{\sum }\limits_{T=1}^{n}({R}_{max}\,\times \,{W}_{T})$$

SHI_cat_ = Social hotspot index for a category

T = Theme (e.g. risk of child labour)

W_T_ = Weight assigned to the theme (1.5 or 1.0)

R_avg_ = Average risk across the theme

R_max_ = Max. possible risk for a theme (all issues v. high).

In this study, we modelled two different CSS for each geographical scenario, one for the relevant feedstock production and a second using the “Chemical, rubber and plastic products” sector as a proxy in the absence of a specific sector for biorefineries, in line with the approach followed in a previous similar study^49^. The main results presented are aggregated SHI results.

#### Integrated cost and environmental assessment

We carried out anticipatory cost and environmental assessment using an integrated modelling framework developed specifically for this study called SustAssessR (Supplementary Fig. 1, code available at: https://doi.org/10.5281/zenodo.3560207). All modelling was undertaken using the R statistical programming language^54–62^.

We developed the process model for cadaverine and putrescine production by building on a previously reviewed process from Kind *et al*.^63^; this model involves fermentation followed by downstream processing and work-up through centrifugation, solvent extraction, and distillation (Supplementary Fig. 2). We added process steps for handling of excess biomass and waste with two versions of the process model to reflect different waste handling scenarios:

- Integrated: Waste cake burned in the combustor, yielding process steam.
- Non-integrated: Waste cake sent for incineration (modelled as municipal incineration).

#### Model parameterisation

We determined the stoichiometric yield trade-off between biomass and product per glucose via flux balance analysis using the *E. coli* genome scale model iML1515^64^. We used the stoichiometric outputs as inputs for an in-house built fermentation model utilizing mass balance first principles. The fermentation model used common fermentation conditions for the host organism to simulate key performance indicators such as titre, productivity, and yield and we simulated several scenarios, including different product yields, organism uptake rates, and time switches between growth of biomass and product formation. The results inferred raw material requirements, such as sugar and nitrogen, and were used for determining the downstream material flows for a plant with an output of 100 kilotonnes per year. For the solvent extraction step, we assumed solvent load requirement based on information from the literature^65^.

With the exception of the distillation steps, we derived steam and electricity requirements of key process steps from the BREW project generic approach^66^. We modelled the heat required for distillation of compounds as the sum of the theoretical sensible heat required to raise the temperature of the compound to its boiling point and the enthalpy of vaporization, all divided by an estimated distillation efficiency^67^:

$${E}_{heat}=(c\Delta T+\,\Delta {H}_{Vap})/Ef{f}_{Dist}\,$$

c = sensible heat

∆T = change in temperature

∆H_Vap_ = enthalpy of vaporisation

Eff_Dist_ = efficiency of distillation.

This is a similar approach to that taken in BREW project’s “generic approach”, but now including sensible heat and a more conservative efficiency consideration^9^.

#### Uncertainty propagation

We represented parameters with probability distributions to account for uncertainty. Uncertainty distributions were derived from published ranges of values where possible. Where only single figures could be found, we took a conservative approach, constructing a triangular distribution with the published figure as the modal value and maximum and minimum values corresponding to double and/or half the published figure. To propagate the uncertainty, we employed a Monte Carlo approach with 10,000 iterations using pseudorandom variables to sample from the specified uncertainty distributions. Scenario uncertainty (e.g. waste handling, geographical location/feedstock, energy source, nitrogen source) was also propagated by sampling from these discrete distributions of possibilities. All parameters and their associated distributions are outlined in the supplementary material (Supplementary Table 7).

#### Life-cycle assessment

We conducted life-cycle assessment (LCA) in line with the ISO standards following an attributional approach and a cradle-to-gate system boundary (Supplementary Fig. 3)^39,40^. Foreground mass and energy flows were derived from the process modelling described above. The primary background data source was Ecoinvent v3.3^68^ database as implemented in the Gabi LCA software^69^. We carried out impact assessment using ReCiPe 2016 under the hierarchist perspective^70^ (although egalitarian and individualist perspectives for cadaverine and putrescine production are also provided in the supplementary information). We calculated climate change impact excluding biogenic carbon dioxide and the applied a credit for carbon dioxide embodied in the product^71^.

The sources and names of background data used in this project are outlined in Supplementary Table 8. Energy source (for steam and electricity) was randomly varied between biomass and grid (electricity grid/natural gas) for each Monte Carlo run. We chose municipal solid waste incineration as the most appropriate proxy for waste treatment, in the absence of data concerning the specific composition of the waste cake generated. In the absence of a specific background dataset for biomass combustion, we chose data for softwood combustion as a proxy for combustion to generate heat due to its similar water content. In line with NREL modelling, we assumed evaporation to be effective at reducing water content to 60%^72^. The construction of the fermentation plant was taken into account using Ecoinvent v3.3 data for a bioethanol fermentation plant scaled according to the number of fermenters required as determined in the process model.

We modelled our different feedstocks as described previously (Supplementary Table 9). Data for sucrose from sugarcane (Brazil) and sucrose from sugar beets (France) was sourced from Ecoinvent v3.3. For the production of corn starch and corn stover, we used data from the US Life Cycle Inventory (LCI) Database^73^. For processing of corn starch, we used an LCI from Renouf *et al*. (Supplementary Table 10)^74^, while we sourced corn stover processing data from the NREL 2017 sugars model^75^ and the corresponding 2015 report for emissions data (Supplementary Table 11)^76^.

We wanted to identify key hotspots and sensitivities, thus, we first calculated results for the production of cadaverine and putrescine monomers where the functional unit was 1 kg of monomer production. To allow comparisons between monomers in their polyamide context, we considered four different polyamide usage scenarios (nylon 66, nylon 46, nylon 410, nylon 510; see Fig. 5A); we compared these on a “like-for-like” mass basis due to their generally comparable physical properties^29^. For such cases, the functional unit was 1 kg of nylon. The data sources for each of the monomers are provided in Supplementary Table 12. We sourced an LCI for fossil-based HMDA production from published literature and adapted it with global scale background data from Ecoinvent v3.3 (Supplementary Table 13)^68,77^. We used data from thinkstep for sebacic acid production from castor bean and from Ecoinvent 3.3 for fossil-based adipic acid production^68,69^. For the climate change impact of adipic acid production, we randomly varied the value selected for each Monte Carlo run between the Ecoinvent v3.3 value (assuming 80% N_2_O abatement) and a sensitivity case (assuming 98% N_2_O abatement) as modelled by Aryapratama *et al*.^78^. This takes account of variability in the N_2_O abatement strategies of the incumbent production process.

We derived the steam and electricity requirements of nylon 66 manufacture from the Plastics Europe ecoprofile for all nylon types as preparation of nylons using adipic acid and sebacic acid occurs under similar conditions^28,79^. We assumed that the polymerisation site was located relatively close to monomer production (within the same country/state) and so the transportation distance was modelled accordingly as a uniform distribution between 100 and 400 km. Full LCIs at unit-process and aggregated level are provided in Supplementary Tables 14 and 15. Life-cycle impact assessment (LCIA) results for monomer and nylon production are provided in Supplementary Tables 16 and 17. Hotspot results for monomer production are provided in Supplementary Table 18.

#### Minimum selling price calculation

We calculated the minimum selling price (MSP) as the minimum price needed to make the net present value (NPV) of the project zero over its lifespan. We assumed a minimum acceptable rate of return (and therefore discount rate) of between 10 and 24%. The lower figure was chosen as an industry-standard while the higher figure reflects the high-risk nature of the project^76,80^. We calculated the relative contribution of different cost elements using the methodology outlined in Supplementary Fig. 4 with economic assumptions guided by the literature, NREL models, and the BREW project (Supplementary Table 19)^66,76,80,81^. We determined prices and costs from a range of sources with a decision hierarchy that guided this process (Supplementary Table 20). The distributions used are outlined in Supplementary Table 21 ^76,80,82^; all figures are in 2014 US$ due to data availability constraints. Capital cost was estimated based on a published figure for an advanced biorefinery^82^. This figure, $149 million in 2011 for a 33 kilotonne biorefinery, was scaled to 100 kilotonnes using a scaling exponent of 0.836^81^ and the CEPCI index to convert to 2014 US$. The cost was then scaled using the same exponent according to the number of fermenters required as determined in the process model.

We fully integrated the MSP model with the process model and life-cycle assessment described above. The model integrated the outputs from the process model with uncertain parameters pertaining to economic and cost assumptions. We annualised the capital cost to a capital charge using a similar approach to the aforementioned calculation of the minimum selling price whereby the charge was set at a level which would make the NPV of the capital investment zero at the end of the project.

#### Sensitivity test

To test sensitivity of the model to individual parameters and their relative influence on the results we varied each parameter individually throughout its range while holding all others steady to determine which parameters were most influential for the final outcomes. We employed a multi-start methodology to take into account how individual parameter influence might vary across the parameter space^83^. For each parameter investigated, we re-ran the analysis (with 1000 iterations) starting in different regions of the parameter space each time. We selected the starting location at random based on the specified probability distributions described previously. Results of the sensitivity analysis are provided in Supplementary Table 22.

### Interpretation

The *interpretation* stage centred around a second set of 6 workshops involving 32 company employees across various teams (Supplementary Table 6). In advance of the workshop, we circulated a short summary sheet to all participants along with a detailed report of results. The results reported in this article represented a slightly updated version of what was presented to stakeholders reflecting the iterative and continuous nature of the process. However, the key implications and conclusions have not changed. N.E.M. facilitated all workshops. At the start of the workshops, N.E.M. made a short (~10 minute) presentation of results. Subsequently, the following topics were discussed:

- Discussion of results: What do you think of the results? Are they as expected? Were there any unexpected results?
- Making decisions: How could the results be used? Do they change how you might make decisions?
- Future work: Where do we need more information and clarity? What are the priorities for further analysis and data collection?

For wider engagement of internal stakeholders, N.E.M. also presented results and project context at an hour-long, company-wide internal seminar. We then electronically circulated a summary report and survey. In the survey participants were asked:

- What they thought of the results of the assessment.
- What impact the results had on the way they think about the sustainability of bio-based processes.
- What they thought about the application of frameworks like CSA in the biotechnology industry.

Results of these engagements were coded and analysed in Nvivo 12^84^ to identify emergent themes.

## Supplementary information

Supplementary information

Supplementary Dataset 14

Supplementary Dataset 15

Supplementary Dataset 16

Supplementary Dataset 17

Supplementary Dataset 18

Supplementary Dataset 22

Supplementary Dataset 23

## Data Availability

The methods and supplementary materials provide information on all data sources used. All outputs of the analysis are provided in the supplementary material except intermediate fermenter modelling outputs which are proprietary. However, the procedures used to calculate these outputs are disclosed in the methods and all final outputs of the analysis are included in the article.
