# Supplementary material for: Collaborating constructively for sustainable biotechnology
Source: Sci Rep. 2019 Dec 13;9:19033. doi: 10.1038/s41598-019-54331-7 (PMC6910968; doi:10.1038/s41598-019-54331-7)
Supplement: Supplementary file 1 — Supplementary information [file 41598_2019_54331_MOESM1_ESM.pdf]

Collaborating constructively for sustainable biotechnology

Nicholas E Matthews, Carrie A Cizauskas, Donovan S Layton, Laurence Stamford, Philip Shapira

Supplementary information

Supplementary Figure 1: Program structure for SustAssessR.

Supplementary Figure 2: Process model for cadaverine and putrescine production adapted from Kind and Wittman (2011).

Supplementary Figure 3: System boundary for monomer production.

Supplementary Figure 4: Summary of methodology for calculating total biorefinery costs.

Supplementary Table 1: Summary of different sustainability aspects introduced to the formulation workshops and the formulation survey with their associated average score given in the survey responses to the question: "In your opinion please score the following aspects of sustainability as to how significant they are for the biotechnology sector" (n=153). The relevant SDGs for each aspect are also listed.

Supplementary Table 2: Summary of Answers given in to the question: "How might the following sources of information influence your perspective of the sustainability of a product?" (n=137)

Supplementary Table 3: The CSS used to identify potential social risks/hotspots in the SHDB for each of the four production scenarios analysed in this report.

Supplementary Table 4: Selected individual indicator results for social category "Health & Safety". Figures derived from the SHDB.

Supplementary Table 5: Selected individual indicator results for social category "Labor Rights & Decent Work". Figures derived from the SHDB.

Supplementary Table 6: Deliberative engagement activities undertaken.

Supplementary Table 7: Parameterisations used to generate the process model.

Supplementary Table 8: Background data sources used in the environmental assessment.

Supplementary Table 9: Feedstock scenarios and their corresponding LCI data sources for agricultural production and processing to sugar.

Supplementary Table 10: LCI table of inputs for 1kg sugar production from harvested corn. Figures derived from Renouf et al. (2008).

Supplementary Table 11: LCI for processing of corn stover to sugar (glucose and xylose). Figures derived from two different NREL studies using a consistent base model. Background data sources are outlined in Supplementary Table 9.

Supplementary Table 12: Nylon types considered in analysis and their corresponding data sources.

Supplementary Table 13: Life-cycle inventory for HMDA production with associated Ecoinvent v3.3 dataset used for background data. Data marked with a * denotes cut-off flows which were not modelled. Figures derived from Dros et al. (2015).

Supplementary Table 14: LCI at unit process level for cadaverine and putrescine production (separate excel file).

Supplementary Table 15: Aggregated LCI for cadaverine and putrescine production (separate excel file).

Supplementary Table 16: LCIA results for cadaverine and putrescine production (separate excel file).

Supplementary Table 17: Comparative LCIA results for different nylon types (separate excel file).

Supplementary Table 18: Hotspot results for cadaverine and putrescine production showing relative contributions of different process stages and background data (separate excel file).

Supplementary Table 19: Modelling parameters/assumptions used for costings model. Further details and full citations are provided in the methods.

Supplementary Table 20: Decision hierarchy for determining distributions for prices and costs.

Supplementary Table 21: Summary of price parameterisations used for costings model.

Supplementary Table 22: Full summary of MSP and cost breakdown results (separate excel file).

Supplementary Table 23: Sensitivity test results (separate excel file).

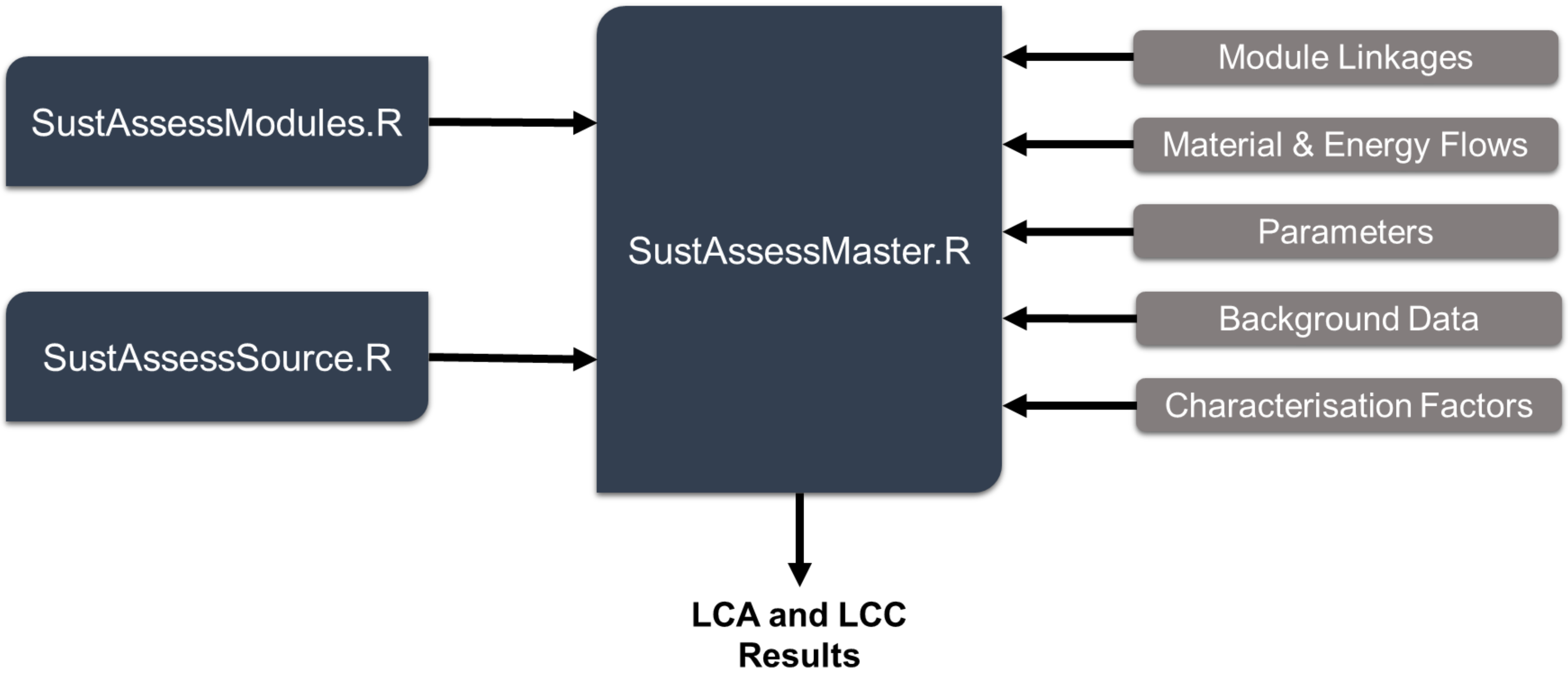

Supplementary Figure 1: Program structure for SustAssessR.

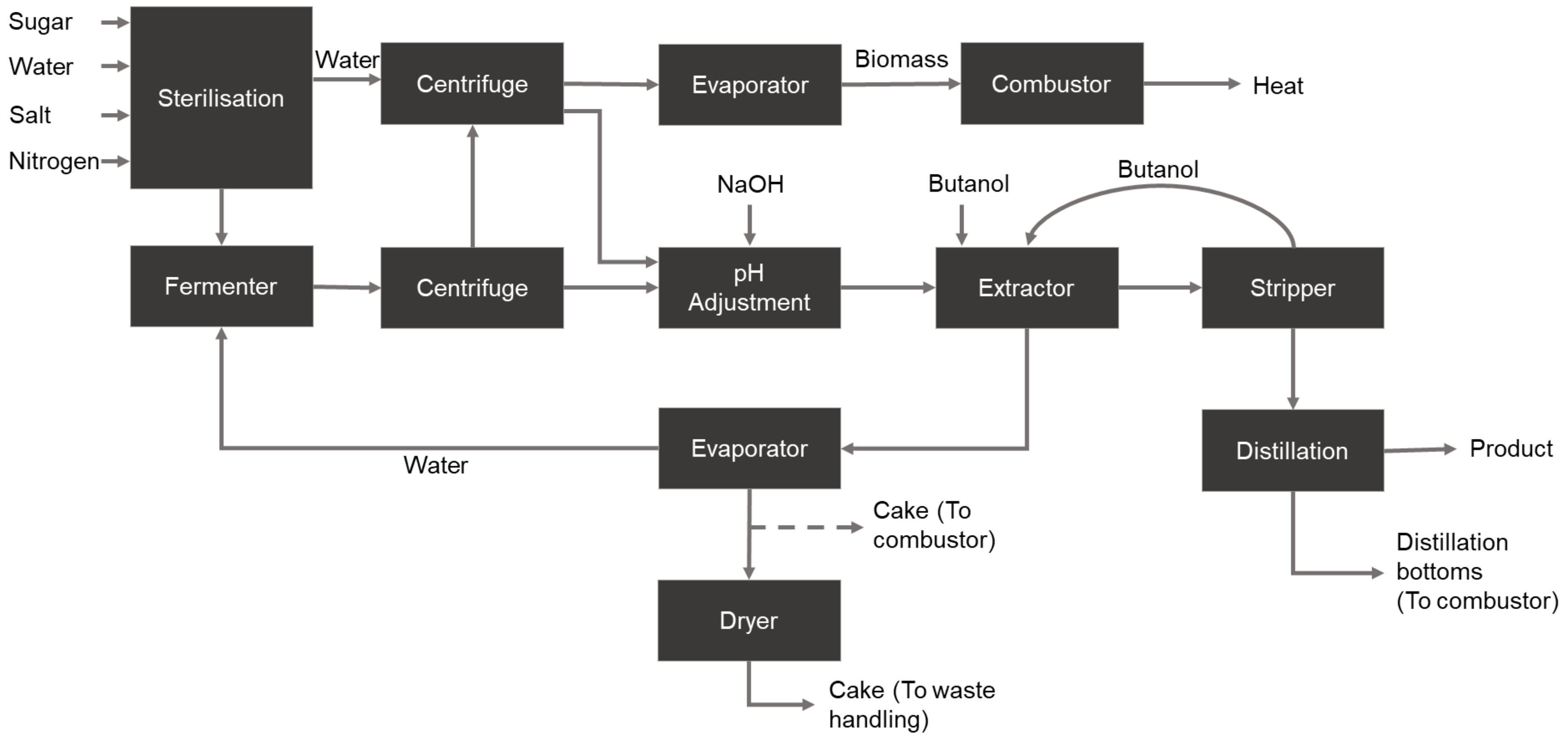

Supplementary Figure 2: Process model for cadaverine and putrescine production adapted from Kind and Wittman 2011.

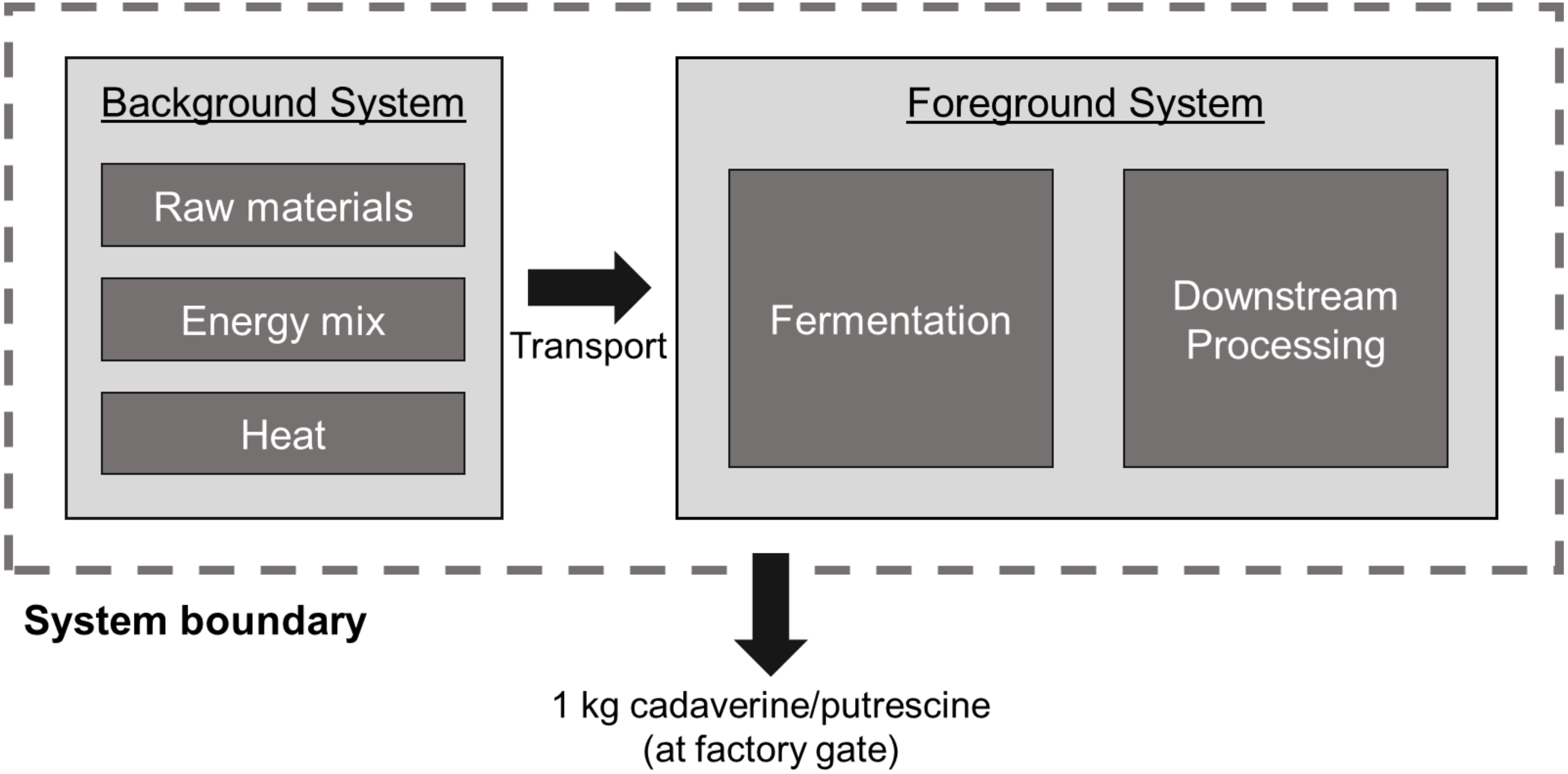

Supplementary Figure 3: System boundary for monomer production.

Total capital investment (TCI) = biorefinery cost * ((# of fermenters/10) ^ scaling exponent (capital))

Raw materials = process model flows * prices

Utilities = process model flows * prices

Waste = process model flows * prices

Total labour = labour cost * ((# of fermenters/10) ^ scaling exponent (labour))

Maintenance = TCI * maintenance rate

Tax = TCI * tax rate

R&D and marketing costs = (total labour + tax + maintenance) * R&D and marketing rate

Overheads = total labour* overheads rate

Other fixed operating costs = maintenance + tax + R&D and marketing + overheads

Supplementary Figure 4: Summary of methodology for calculating total biorefinery costs.

Sustainability Aspect	Average Score (1-5)	Relevant SDGs
Tackling climate change	4.17	15
Improving the health of global ecosystems	4.16	13,14
Promoting equality, peace, and justice	2.99	4,5,10,16
Eliminating poverty, hunger, and poor-health	3.90	1,2,3,6,7
Sustaining employment and economic growth	3.79	8,9,11

Supplementary Table 1: Summary of different sustainability aspects introduced to the formulation workshops and the formulation survey with their associated average score given in the survey responses to the question: "In your opinion please score the following aspects of sustainability as to how significant they are for the biotechnology sector" (n=153). The relevant SDGs for each aspect are also listed.

Influence	Modeling data	Real-world data	Experts	Impacted stakeholders	Civil society organisations	Government resources
Not influential at all	2.19%	0.00%	1.46%	12.41%	7.30%	5.11%
Slightly influential	40.15%	6.57%	10.95%	38.69%	40.88%	37.96%
Quite influential	46.72%	31.39%	59.85%	27.01%	44.53%	43.80%
Very influential	10.95%	62.04%	27.74%	21.90%	7.30%	13.14%

Supplementary Table 2: Summary of Answers given in to the question: "How might the following sources of information influence your perspective of the sustainability of a product?" (n=137)

Production Scenario	CSS Feedstock	CSS Biorefinery
BR Sugarcane	Sugar cane, sugar beet	Chemical, rubber, plastic products
FR Sugar Beet	Sugar cane, sugar beet	Chemical, rubber, plastic products
US Corn	Cereal grains nec	Chemical, rubber, plastic products
US Ligno	Cereal grains nec	Chemical, rubber, plastic products

Supplementary Table 3: The CSS used to identify potential social risks/hotspots for each of the four production scenarios analysed in this report.

Social Theme	Indicator	Brazil chemicals	Brazil sugarcane	France chemicals	France sugar beet	US chemicals	US cereals
Occupation injuries and deaths	Fatal injury rate by country	High	High	Medium	Medium	Medium	Medium
	Fatal injury rate by sector	Very High	Very High	Medium	High	Medium	Very High
	Non-fatal injury rate by country	Very High	Very High	Very High	Very High	Low	Low
	Non-fatal injury rate by sector	Very High	High	Very High	Very High	Medium	Medium
Occupational toxins & hazards	Overall risk of loss of life years by exposure to carcinogens in occupation	Medium	Medium	High	High	Medium	Medium
	Overall risk of workplace noise exposure, both genders	Medium	Medium	Low	Low	Low	Low
	Risk of loss of life years by airborne particulates in occupation	High	High	Medium	Medium	Low	Low

Supplementary Table 4: Selected individual indicator results for social category “Health & Safety”. Figures derived from the SHDB.

Social Theme	Indicator	Brazil Chemicals	Brazil Sugarcane	France Chemicals	France Sugar Beet	US Chemicals	US Cereals
Child labour	Risk of child labour in sector, Total (Qual)	Medium	Medium	No evidence	No evidence	Low	Medium
	Risk of child labour in sector, Total (Quant)	Medium	Very High	No data	No data	No data	No data
Forced Labour	Risk of forced labour by sector	High	Very High	Low	Low	Medium	Medium
Collective bargaining	Risk that country lacks or does not enforce Collective Bargaining rights	Medium	Medium	Medium	Medium	Very High	Very High
	Risk that country lacks or does not enforce Freedom of Association rights	High	High	Medium	Medium	High	High
	Risk that country lacks or does not enforce the right to strike	Medium	Medium	Medium	Medium	High	High
Labour laws	Risk that country does not provide adequate labour laws by sector	Medium	Low	Low	Low	Low	Low
	Risk that country does not ratify ILO conventions by sector	Low	High	Low	Medium	Medium	Medium
	Risk that minimum wage has not been updated	Low	Low	Low	Low	Medium	Medium
Migrant Workers	Risk that migrant workers are treated unfairly (qualitative)	Medium	Medium	Low	Low	Medium	Medium
	Risk that women are not accepted into the country as immigrants	Medium	Medium	Low	Low	Medium	Medium
	Risk that country does not pay immigrants enough for remittances	Medium	Medium	Medium	Medium	High	High
Poverty	Risk of wages being under $2 per day	Medium	Medium	Low	Low	Low	Low
Unemployment	Risk of unemployment in Country	Medium	Medium	High	High	High	High
Wage assessment	Risk of sector average wage being lower than the country's minimum wage	Low	Very High	Low	Low	Low	High
	Risk of sector average wage being lower than the country's non-poverty guideline	Low	Very High	Low	Very High	Low	Medium
Working time	Risk of excessive working time by sector	Low	Low	Medium	Medium	Medium	Medium

Supplementary Table 5: Selected individual indicator results for social category “Labor Rights & Decent Work”. Figures derived from the SHDB.

Date	Activity	CSA Stage	Participants	Number of Participants	Duration	Data collected
August 2018	Workshop	Formulation	Development team	4	1 hour	Notes, wordcloud responses
August 2018	Workshop	Formulation	Legal team	4	1 hour	Notes, wordcloud responses
August 2018	Workshop	Formulation	Business Development team	8	1 hour	Notes, wordcloud responses
August 2018	Workshop	Formulation	Manufacturing team	4	1 hour	Notes, wordcloud responses
August 2018	Survey	Formulation	Company employees	137	N/A	Survey responses
March 2019	Workshop	Interpretation	Development team	5	1 hour	Notes
March 2019	Workshop	Interpretation	Legal team	6	1 hour	Notes
March 2019	Workshop	Interpretation	Business Development team	5	1 hour	Notes
March 2019	Workshop	Interpretation	Modelling team	6	1 hour	Notes
March 2019	Workshop	Interpretation	Products team	5	1 hour	Notes
March 2019	Workshop	Interpretation	Manufacturing team	5	1 hour	Notes
August 2018	Survey	Interpretation	Company employees	54	N/A	Survey responses

Supplementary Table 6: Deliberative engagement activities undertaken.

Parameter	Value	Min	Max	Distribution	Units	Source	Uncertainty Source
**Energy Sterilisation**	0.1	0.1	0.1	Triangular	kg/kg	Patel et al. 2006	Patel et al. 2006
**Energy Agitation Aeration**	3	1	5	Triangular	kwh/m3	Patel et al. 2006	Patel et al. 2006
**Energy Centrifugation**	7	3.5	16	Triangular	kwh/m3	Patel et al. 2006	Patel et al. 2006
**Drying Steam**	1.5	0.95	1.67	Triangular	kg/kg	Patel et al. 2006	Patel et al. 2006
**Evaporation Triple Effect (Steam)**	0.4	0.3	0.5	Triangular	kg/kg	Patel et al. 2006	Patel et al. 2006
**Water Content Biomass**	1.5	1.5	1.5	None	kg/kg	Davis et al. 2013	N/A
**Water Content Waste**	0.3	0.3	0.3	None	kg/kg	Ecoinvent v3.3	N/A
**Biomass to Heat**	14.32	7.16	14.32	Triangular	MJ/kg	Ecoinvent v3.3	Default
**Annual operating time**	7900	7900	7900	None	hours	Industry standard	N/A
**Down time**	12	6	24	Triangular	hours	Assumed	Default
**Solvent required**	0.1	0.05	0.2	Triangular	kg/kg	Krzyzaniak et al. 2013	Default
**Solvent loss rate**	1	0.5	2	Triangular	% per cycle	Assumed	Default
**Distillation efficiency**	23.8	11.9	47.6	Triangular	%	Cavaletto 2013	Default
**Polymerisation electricity**	2.7	2.7	5.4	Triangular	MJ/kg	Plastics Europe 2014	Default
**Polymerisation heat**	6.6	6.6	13.2	Triangular	MJ/kg	Plastics Europe 2014	Default
**Polymerisation transport**	0.2	0.1	0.4	Triangular	tkm/kg	Assumed	Default

Supplementary Table 7: Parameterisations used to generate the process model. Further details and full citations are provided in the methods.

Background Data	Data source	Dataset name	Geographic specificity	Used in
Electricity - grid	Ecoinvent v3.3	Market for electricity, medium voltage	BR/FR/US	General
Electricity - biomass	Ecoinvent v3.3	Heat and power co-generation, wood chips, 6667 kW, state-of-the-art 014	BR/FR/US	General
Heat - grid	Ecoinvent v3.3	Steam production in chemical industry	RoW	General
Heat - biomass	Ecoinvent v3.3	Heat and power co-generation, wood chips, 6667 kW, state-of-the-art 014	BR/FR/US	General
Waste treatment	Ecoinvent v3.3	Treatment of municipal solid waste, incineration	RoW	General
Combustion	Ecoinvent v3.3	Heat production, softwood chips from forest, at furnace 5000kW, state-of-the-art 2014	CH (with GLO background data)	General
Water	Ecoinvent v3.3	Market for water, decarbonised, at user	GLO	General
Sodium Hydroxide	Ecoinvent v3.3	Market for sodium hydroxide, without water, in 50% solution state	GLO	pH adjustment, corn stover processing
Sodium Chloride	Ecoinvent v3.3	Market for sodium chloride, powder	GLO	Fermenter
Ammonium Sulfate	Ecoinvent v3.3	Market for ammonium sulfate, a N	GLO	Fermenter, corn stover processing
Butanol	Ecoinvent v3.3	Market for 1-butanol	GLO	Extraction
Corn Steep Liquor	USLCI	Corn steep liqour	RNA	Corn stover processing
SO2	Ecoinvent v3.3	Market for sulfur dioxide	RoW	Corn stover processing
Soybean Oil	Ecoinvent v3.3	Market for soybean oil, refined	GLO	Corn stover processing
Ammonia	Ecoinvent v3.3	Market for ammonia, liquid	RoW	Corn stover processing
Lime	Ecoinvent v3.3	Market for lime	GLO	Corn stover processing
Sulfuric Acid	Ecoinvent v3.3	Market for sulfuric acid	GLO	Corn stover processing
Fermentation plant	Ecoinvent v3.3	Market for ethanol fermentation plant	GLO	General
Transport	Ecoinvent v3.3	Market for transport, freight, lorry, unspecified	GLO	Polymerisation

Supplementary Table 8: Background data sources used in the environmental assessment

Feedstock Scenario	Location	Raw Feedstock	Sugar	Agricultural data	Processing data
BR Sugarcane	Brazil	Sugarcane	Sucrose	Ecoinvent v3.3	Ecoinvent v3.3
FR Sugar Beet	France	Sugar beets	Sucrose	Ecoinvent v3.3	Ecoinvent v3.3
US Corn	United States	Corn starch	Glucose	US LCI/NREL	Renouf et al. 2008
US Ligno	United States	Corn stover	Glucose and Xylose	US LCI/NREL	NREL

Supplementary Table 9: Feedstock scenarios and their corresponding LCI data sources for agricultural production and processing to sugar.

Input	Amount	Unit	Data Source (Ecoinvent v3.3)
Corn	1.50E+00	kg	RNA: Corn, production, average, US, 2022
Electricity	9.34E-01	MJ	US: market group for electricity, medium voltage
Natural Gas	1.66E-01	m3	US: market for natural gas, high pressure
Chlorine	1.20E-05	kg	GLO: market for chlorine, liquid
Cyclohexane	5.50E-05	kg	GLO: market for cyclohexane
Lime	3.00E-04	kg	GLO: market for lime
Sodium chloride	6.50E-05	kg	GLO: market for sodium chloride, powder
Sodium hydroxide	2.82E-04	kg	GLO: market for sodium hydroxide, without water, in 50% solution state
Sulfur dioxide	3.06E-03	kg	RoW: market for sulfur dioxide, liquid
Sulfuric acid	4.50E-04	kg	GLO: market for sulfuric acid
Urea	2.08E-04	kg	GLO: market for urea, as N

Supplementary Table 10: LCI table of inputs for 1kg sugar production from harvested corn. Figures derived from Renouf et al. (2008).

Inputs	Amount	Units	Source
Corn Stover	1.04E+05	kg	NREL 2017 Biochemical Sugar Model
Sulfuric Acid	2.24E+03	kg	NREL 2017 Biochemical Sugar Model
NaOH	1.42E+03	kg	NREL 2017 Biochemical Sugar Model
Ammonia	6.82E+02	kg	NREL 2017 Biochemical Sugar Model
Glucose	1.21E+03	kg	NREL 2017 Biochemical Sugar Model
Corn Steep Liqour	8.20E+01	kg	NREL 2017 Biochemical Sugar Model
Corn Oil (Modelled as soybean oil)	7.00E+00	kg	NREL 2017 Biochemical Sugar Model
Water	1.99E+05	kg	NREL 2017 Biochemical Sugar Model
Lime	1.51E+02	kg	NREL 2017 Biochemical Sugar Model
SO2	8.00E+00	kg	NREL 2017 Biochemical Sugar Model
Host Nutrients (Ammonium Sulfate)	3.40E+01	kg	NREL 2017 Biochemical Sugar Model
**Outputs**			
Glucose	3.03E+04	kg	Davis et al. 2015
Xylose	1.67E+04	kg	Davis et al. 2015
Ash	4.46E+03	kg	NREL 2017 Biochemical Sugar Model
Electricity	1.41E+04	kWh	NREL 2017 Biochemical Sugar Model
**Emissions**			
Carbon Dioxide	7.47E+04	kg	Davis et al. 2015
Methane	1.60E+00	kg	Davis et al. 2015
Nitrogen dioxide	5.30E+01	kg	Davis et al. 2015
Carbon monoxide	5.30E+01	kg	Davis et al. 2015
Sulfur dioxide	1.10E+01	kg	Davis et al. 2015

Supplementary Table 11: LCI for processing of corn stover to sugar (glucose and xylose). Figures derived from two different NREL studies using a consistent base model. Background data sources are outlined in Supplementary Table 9.

Nylon Type	Diamine		Dicarboxylic Acid	
	Name	LCI Data Source	Name	LCI Data Source
Nylon 6,6	HMDA	Dros et al. 2015	Adipic acid	Ecoinvent v3.3
Nylon 4,6	Putrescine	This study	Adipic acid	Ecoinvent v3.3
Nylon 4,10	Putrescine	This study	Sebacic acid	thinkstep
Nylon 5,10	Cadaverine	This study	Sebacic acid	thinkstep

Supplementary Table 12: Nylon types considered in analysis and their corresponding data sources.

Input	Amount	Units	Data source (Ecoinvent v3.3)
HCN	5.43E-01	kg/kg	GLO: market for hydrogen cyanide
Butadiene	5.45E-01	kg/kg	GLO: market for butadiene
NH3	1.75E-03	kg/kg	RoW: market for ammonia, liquid
Steam	8.46E+00	kg/kg	GLO: market for steam, in chemical industry
Electricity	4.60E-01	kwh/kg	GLO: market group for electricity, medium voltage
Fe-catalyst*	6.00E+00	g/kg	N/A
Hydrogen	6.70E-02	kg/kg	RoW: market for hydrogen, liquid
Sodium bisulfite*	1.09E-01	kg/kg	N/A
Sodium sulfite	6.70E-02	kg/kg	GLO: market for sodium sulfite
Process water	1.23E+00	kg/kg	GLO: market for water, decarbonised, at user
Inert gas*	1.00E-02	L/kg	N/A
Cooling water*	2.72E-01	m3/kg	N/A

Supplementary Table 13: Life-cycle inventory for 1kg HMDA production with associated Ecoinvent v3.3 dataset used for background data. Data marked with a * denotes cut-off flows which were not modelled. Figures derived from Dros et al. (2015).

Name	Distribution	Units	Mode	Min	Max	Source
**Tax Rate**	Uniform	%	N/A	0.7	3.0	Davis et al. 2015 (Min)<br>Gargalo et al. 2016 (Max)
**Maintenance Rate**	Uniform	%	N/A	3.0	6.0	Davis et al. 2015 (Min)<br>Gargalo et al. 2016 (Max)
**R&D and Marketing Costs**	Triangular	%	6.0%	3.0	12.0	Patel et al. 2006 (Mode)<br>Default uncertainty
**Overheads Rate**	Uniform	%	N/A	60.0	90.0	Gargalo et al. 2016 (Min)<br>Davis et al. 2015 (Max)
**Interest Rate**	Triangular	%	8.0	4.0	16.0	Davis et al. 2015 (Mode)<br>Default uncertainty
**Income Tax Rate**	none	%	35.0	N/A	N/A	Davis et al. 2015
**Labour Scaling Factor**	none	exponent	0.25	N/A	N/A	Patel et al. 2006
**Capital Scaling Factor**	none	exponent	0.836	N/A	N/A	Gallagher et al. 2006
**Discount Rate**	Uniform	%	N/A	10.0	24.0	Davis et al. 2015 (Min)<br>Gargalo et al. 2016 (Max)
**Loan Repayment Period**	none	years	10	N/A	N/A	Davis et al. 2015

Supplementary Table 19: Modelling parameters/assumptions used for costings model. Further details and full citations are provided in the methods.

Grade	Modal Value	Uncertainty range
1st (Best)	Average from historic price trend or literature figure	Historic price trend (specific) or literature figure
2nd	Estimate from literature/industry	Historic price trend (generic, US Gov)
3rd	Estimate from literature/industry	Historic price trend (generic, Index Mundi)
4th (Worst)	Estimate from literature	Generic estimate (double and half)

Supplementary Table 20: Decision hierarchy for determining distributions for prices and costs.

Parameter	Distribution	Value	Min	Max	Units	Figure Source	Uncertainty Source	Grade
**Sugar**	Triangular	0.37	0.20	0.65	$/kg	Sugar #11[1]	Sugar #11[1]	1st
**Lignocellulosic Sugar**	Triangular	0.41	0.20	0.81	$/kg	NREL[2]	Default	4th
**Butanol**	Triangular	1.56	0.97	2.20	$/kg	Gargalo et al. 2016	Gargalo et al. 2016	1st
**Salt**	Triangular	0.06	0.04	0.09	$/kg	USGS[3]	IndexMundi[4]	3rd
**Ammonium Sulfate**	Triangular	0.59	0.42	0.66	$/kg	USDA[5]	USDA[5]	1st
**Ammonium Nitrate**	Triangular	0.62	0.44	0.69	$/kg	USDA[5]	USDA[5]	1st
**Corn Steep Liqour**	Triangular	0.08	0.05	0.08	$/kg	Davis et al. 2015	USDA[5]	2nd
**Sodium Hydroxide**	Triangular	0.20	0.14	0.22	$/kg	Davis et al. 2015	IndexMundi[4]	3rd
**Electricity**	Triangular	0.07	0.06	0.07	$/kWh	EIA[7]	EIA[7]	1st
**Steam**	Triangular	0.44	0.15	0.64	¢/kg	Gargalo et al. 2016	IndexMundi[8]	3rd
**Water**	Triangular	0.05	0.03	0.07	¢/kg	Gargalo et al. 2016	IndexMundi[4]	3rd
**Wastewater treatment**	Triangular	0.05	0.03	0.11	¢/kg	Gargalo et al. 2016	Default	4th
**Waste management**	Triangular	0.04	0.02	0.07	$/kg	Gargalo et al. 2016	Default	4th
**Capital Cost of Plant**	Triangular	370.28	185.14	740.56	m$	Tsagkari et al. 2016	Default	4th
**Labour Cost**	Triangular	3.66	1.83	7.32	m$/yr	Davis et al. 2015	Default	4th

Supplementary Table 21: Summary of price parameterisations used for costings model.

[1] *https://www.indexmundi.com/commodities/?commodity=sugar&months=120*
[2] *https://www.nrel.gov/extranet/biorefinery/aspen-models/downloads/bc1707a/sugar-model-readme.pdf*
[3] *https://minerals.usgs.gov/minerals/pubs/commodity/salt/mcs-2015-salt.pdf*
[4] *https://www.indexmundi.com/commodities/?commodity=industrial-inputs-price-index&months=120*
[5] *https://www.ers.usda.gov/data-products/fertilizer-use-and-price.aspx*
[7] *https://www.eia.gov/electricity/data.php*
[8] *https://www.indexmundi.com/commodities/?commodity=energy-price-index&months=180*
